# Fitness of Transgenic Mosquito *Aedes aegypti* Males Carrying a Dominant Lethal Genetic System

**DOI:** 10.1371/journal.pone.0062711

**Published:** 2013-05-14

**Authors:** Blandine Massonnet-Bruneel, Nicole Corre-Catelin, Renaud Lacroix, Rosemary S. Lees, Kim Phuc Hoang, Derric Nimmo, Luke Alphey, Paul Reiter

**Affiliations:** 1 Unité Insectes et Maladies Infectieuses, Institut Pasteur, Paris, France; 2 Oxitec Ltd, Oxford, United Kingdom; 3 Department of Zoology, University of Oxford, Oxford, United Kingdom,; Kansas State University, United States of America

## Abstract

OX513A is a transgenic strain of *Aedes aegypti* engineered to carry a dominant, non-sex-specific, late-acting lethal genetic system that is repressed in the presence of tetracycline. It was designed for use in a sterile-insect (SIT) pest control system called RIDL® (Release of Insects carrying a Dominant Lethal gene) by which transgenic males are released in the field to mate with wild females; in the absence of tetracycline, the progeny from such matings will not survive. We investigated the mating fitness of OX513A in the laboratory. Male OX513A were as effective as Rockefeller (ROCK) males at inducing refractoriness to further mating in wild type females and there was no reduction in their ability to inseminate multiple females. They had a lower mating success but yielded more progeny than the wild-type comparator strain (ROCK) when one male of each strain was caged with a ROCK female. Mating success and fertility of groups of 10 males—with different ratios of RIDL to ROCK—competing for five ROCK females was similar, but the median longevity of RIDL males was somewhat (18%) lower. We conclude that the fitness under laboratory conditions of OX513A males carrying a tetracycline repressible lethal gene is comparable to that of males of the wild-type comparator strain.

## Introduction

Dengue is the most important arbovirus transmitted by mosquitoes; 2.5 billion people live in areas at risk of epidemic transmission [1], [2]. The principal urban vector of dengue, yellow fever and chikungunya is *Aedes aegypti*. No vaccines are available for dengue or chikungunya, so mosquito control is the only option for reducing transmission. In recent decades, however, conventional methods of control have proven insufficiently effective [1]–[3] so there is an urgent need for new and innovative strategies.

Transgenic insects are receiving increasing attention for the control of mosquito-borne diseases [4], [5]. Two broad classes of strategy have been proposed [6]: (i) population reduction, for example by variants of Sterile Insect Technique (SIT) [7]–[11] and; (ii) replacement of the wild population by insects that are refractory to pathogens [12], [13]. It is now feasible to create transgenic strains using transposons, fluorescent proteins and tissue- or stage-specific promoters [14], [15], and several species of culicine and anopheline mosquitoes have been transformed [14], [16].

OX513A is a transgenic strain of *Aedes aegypti* engineered to carry a dominant, repressible, non-sex-specific, late-acting lethal genetic system, together with an Act5C-DsRed2 fluorescent marker [17]. It is intended for use in a sterile-insect pest control system called RIDL® (Release of Insects carrying a Dominant Lethal gene or genetic system) [18]. Without tetracycline, larvae carrying one or more copies of the OX513A insertion develop normally but die at pupation. This late-acting lethality has theoretical advantages over the early-acting lethality characteristic of other sterilisation methods (e.g. radiation, chemicals, *Wolbachia*-induced cytoplasmic incompatibility), at least if there are density-dependent effects before the late-lethal phase [17], [19]. If reared in the presence of tetracycline (e.g. 30 µg/ml), the lethal gene is repressed; tetracycline therefore acts as an ‘antidote’ or repressor of the lethal system to allow the RIDL strain to be reared under defined conditions. The proposed strategy [9], [11], [20], [21] is to mass-rear homozygous RIDL *Ae. aegypti* mosquitoes and release males to mate with wild females in the field. Each egg fertilised by a RIDL male carries the transgene and therefore dies; the RIDL males are therefore effectively sterile [11]. If females only mate once, as is generally assumed for *Ae. aegypti*, then the female's entire reproductive output is destroyed by mating to a RIDL male and she is herself therefore effectively sterilised. However, female monogamy is not a requirement of the approach, though where multiple mating is common, post-copulatory effects such as sperm competition are also relevant.

The success of any SIT-like vector control strategy depends on the performance of the organisms released. Assessing parameters of fitness in the laboratory is the first and necessary step before performing any field releases. Marrelli [22] reviewed three studies of fitness in transgenic mosquitoes. Briefly, in cage experiments, Catteruccia [23] showed that the transgenic allele frequency decreased through time, when introduced into mixed cages of transgenics and wild-type insects, in four strains of *Anopheles stephensi* expressing the enhanced green fluorescent protein (EGFP) or DsRed. Irvin [24] found that estimates of survivorship, longevity and fecundity for three strains of *Ae. aegypti* homozygous for transposase genes and EGFP were lower than for the wild strain. However, Marcelo Jacobs-Lorena's lab reported that while a set of transgenic *An. stephensi* lines expressing a bee venom component had significant fitness problems, another set expressing a synthetic peptide did not [25]. Later work showed that such lines could even have a net fitness advantage in certain circumstances, albeit highly artificial ones [26], [27]. The lower fitness observed for homozygous transgenic mosquitoes in some studies could either be due to (i) insertional mutagenesis and/or negative effects of transgene products or (ii) inbreeding and the harmful effects of homozygous recessive genes [22]. The study of Moreira [25] was designed to distinguish between these two hypotheses and their results suggested that transgenesis is not always deleterious if inbreeding is minimised; Allen et al [28] reached a similar conclusion for transgenic *Cochliomyia hominivorax*.

We report on the first laboratory studies of selected fitness parameters for homozygous *Ae. aegypti* RIDL males viz: (i) mating competitiveness between RIDL and ROCK males for ROCK females, (ii) insemination rate and (iii) adult male longevity. The OX513A was originally generated in a ROCK background, thus making this the most suitable wild type comparator strain for assessing the impact of transgenesis on fitness. We also assessed the lethality of the RIDL construct in heterozygous RIDL/wild type progeny reared without tetracycline, i.e. the progeny of wild type females mated with RIDL males. We discuss implications for the suppression of *Ae. aegypti* populations in the field.

## Results

### Fertilisation and oviposition

In the 2 ♂/1 ♀ experiment, all females (n = 146) took a blood meal and 137 (93.8%) laid eggs. Of the 9 that failed to lay eggs, 3 had sperm in 2 of their three spermathecae. Thus, 140/146 (95.8%) of females had been inseminated successfully. In the 10 ♂/5 ♀ experiment, all females (n = 236) took a blood meal and 199 (84.3%) laid eggs. Of the 37 females that failed to lay eggs, 1 (2.7%) (a ROCK control) had no sperm, 32 (86.3%) had sperm in two spermathecae, and 4 (11%) had sperm in all three.

**Figure 1:**
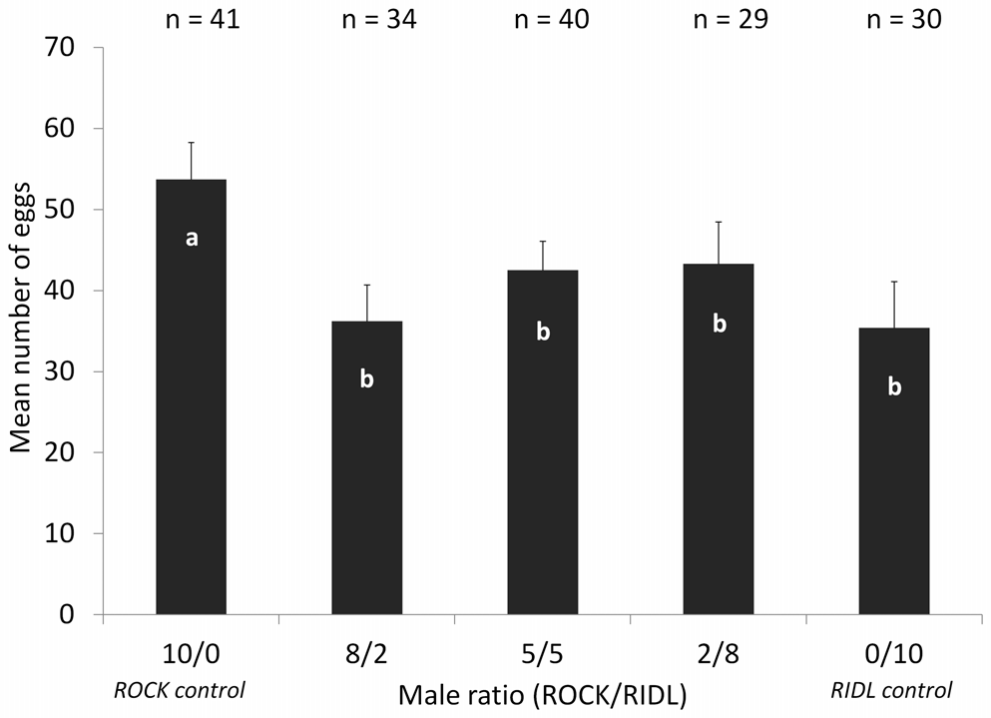
Egg production per female with different proportions of ROCK and RIDL males. For each ratio of strains (ROCK/RIDL): mean number of eggs with Standard Errors (±SE). Above the figure, values (n) indicate the no. of females laying eggs. There is a significant difference in the mean number of eggs across the five ratio of strains, bars with different letters are significantly different (Generalized linear model: p = 0.00561).

In the 2 ♂/1 ♀ experiment, there were more eggs laid in transgenic crosses than in non- transgenic crosses (Table 1; Mann-Witney test, U = 1863, p = 0.043). In the 10 ♂/5 ♀ experiment, there was no significant difference between the number of eggs laid in transgenic crosses than in non transgenic crosses (Table 2; Mann-Witney test, U = 4402, p = 0. 41). There was a significant difference in the number of eggs across all the five different ratios of strains, the ROCK control had a significantly higher number of eggs than the other ratios (Figure 1; GLM, ROCK control: p = 0.00561, other treatments: p>0.05).

**Table 1:** Mating competitiveness experiment 2 ♂/1 ♀.

	Total replicates		Controls		RIDL vs. ROCK	
Mating partner	No. of crosses	Mean no. of eggs	No. of crosses	Mean no. of eggs	No. of crosses	Mean no. of eggs
RIDL male	63	128.0±3.5	48	125.7±4.5	15	135.1±4.2
ROCK male	74	113.7±4.6	41	107.2±6.5	33	122±6.2

For total replicates, the RIDL and ROCK controls and the actual competition experiment (RIDL vs. ROCK), values are given for the number of crosses ( = no. of females laying viable eggs) and for the mean number of eggs laid per female with Standard Errors (±SE). When comparing data from the total replicates, ROCK females fertilised by RIDL males laid more eggs (Mann-Witney, U = 1863, p = 0.043) whereas in the competition experiment (RIDL vs. ROCK), there were fewer observed fertilisations by RIDL males than expected (χ^2^ test, χ^2^ = 6.75, df = 1, p = 0.009).

**Table 2:** Mating competitiveness experiment 10 ♂/5 ♀.

Mating partner	No. of crosses	Mean no. of eggs	No. of males	No. of females	Mean emergence rate
RIDL male	83	54.8±3.1	332	184	14.5±0.02%
ROCK male	114	51.4±2.4	2087	2094	98.3±0.01%

This dataset includes 3 replicates of 6 ROCK/4 RIDL and 4 ROCK/6 RIDL. For transgenic and non transgenic crosses: number of crosses ( = no. of females laying viable eggs), mean no. of eggs laid per female with Standard Error (±SE), total no. of emerging males and females and mean emergence rate (no. of adults/no. of larvae) with Standard Error (±SE). There was no significant difference in the number of eggs laid by females fertilised either by RIDL or ROCK males (Mann-Witney, U = 4402, p = 0. 41).

### Mortality and emergence

**Figure 2:**
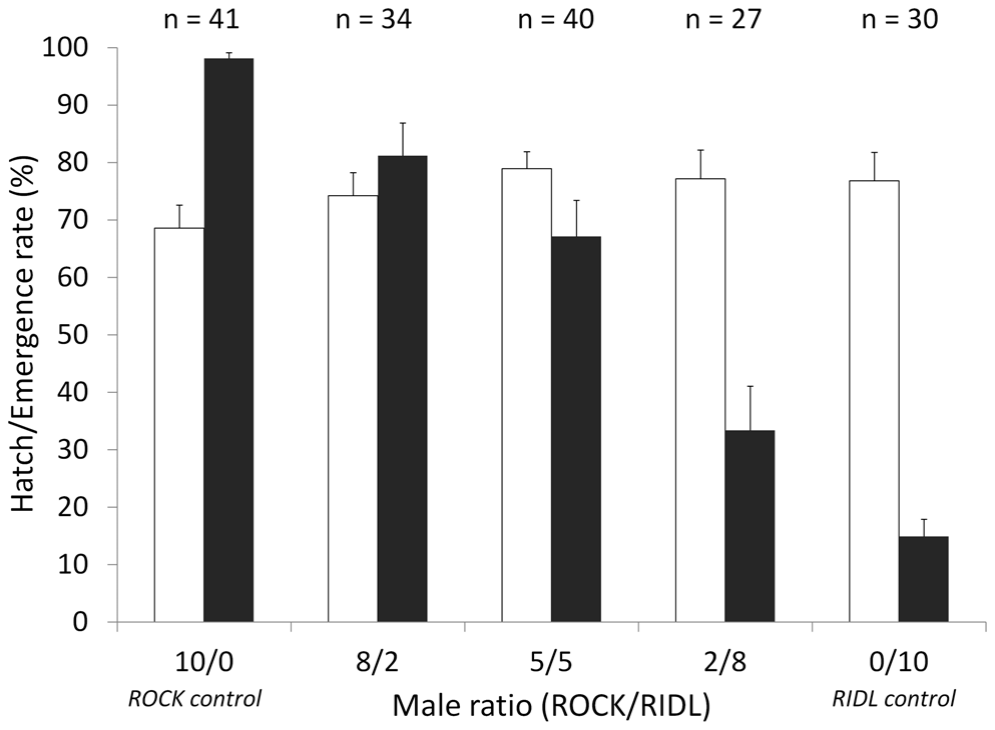
Egg hatch rate for different proportions of ROCK and RIDL males. For each ratio of strains (ROCK/RIDL): mean hatching rate (no. of larvae/no. of eggs, white bars) and mean emergence rate (no. of adults/no. of larvae, black bars) with Standard Errors (±SE). Above the figure, values (n) indicate the no. of females laying viable eggs.

In the 10 ♂/5 ♀ experiment, mean mortality of offspring for all transgenic crosses (n = 83) was 85.5%, i.e. mean adult emergence was 14.5% (Table 2): of the heterozygous RIDL adults collected, 164/496 (33%) were females and 332/496 (67%) were males (Table 2). Overall emergence was the highest for the ROCK control (98.1%) and the lowest for the RIDL control (15%) and, as expected, decreased as the proportion of RIDL males increased (Figure 2).

### Mating competitiveness

In the 2 ♂/1 ♀ experiment, there was a significant deviation from expectation in the observed frequencies of transgenic and non-transgenic matings (Table 1; χ^2^ = 6.75, df = 1, p = 0.009): of the 48 such matings, 15 were transgenic (31%) and 33 were non transgenic (69%).

In the 10 ♂/5 ♀ experiment, there were no significant differences between expected and observed transgenic vs. non-transgenic mating for the different proportions of ROCK/RIDL males (χ^2^ test: 8 ROCK/2 RIDL: χ^2^ = 3.18, df = 1, p = 0.075, n = 34; 5 ROCK/5 RIDL: χ^2^ = 0.9, df = 1, p = 0.343, n = 40 and 2 ROCK/8 RIDL: χ^2^ = 0.012, df = 1, p = 0.912, n = 27). In both mating experiments (n = 336), there was only one case (0.30%) in which both fluorescent and non-fluorescent progeny were observed, which presumably represents a female mating both a RIDL and a ROCK male.

### Insemination rate

When contact was limited to 24 hours, there were no significant differences between the number of females fertilized by RIDL males; 4.90±0.60 females (n = 150, range 1–7) or ROCK males; 5.2±0.32 females (n = 150, range 4–7), χ^2^ = 0.043, df = 1, p = 0.84. The same applied when mosquitoes were kept together for several days. RIDL males fertilised 4.70±0.68 females (n = 150, range 0–8) and ROCK males fertilised 3.6±0.60 females (n = 150, range 0–6), χ^2^ = 1.473, df = 1, p = 0.23. The number of full spermathecae among inseminated females was also not significantly different between strains (For 24 h exposure to males: RIDL: 2.19 (±0.077) vs. ROCK: 2.39 (±0.119), Mann-Whitney U test: U = 16.5, p = 0.1841; for indefinite exposure to males: RIDL: 1.94 (±0.228) vs. ROCK: 1.80 (±0.260), Mann-Whitney U test: U = −5, p = 0.7279). The average number of spermathecae inseminated in the “24 h” experiment was not significantly higher than during the “until death” experiment (Experiment 24 h: 2.28 (±0.072) vs. Experiment until death: 1.87 (±0.169), Mann-Whitney U test: U = −65, p = 0.0646).

### Adult male longevity

**Figure 3:**
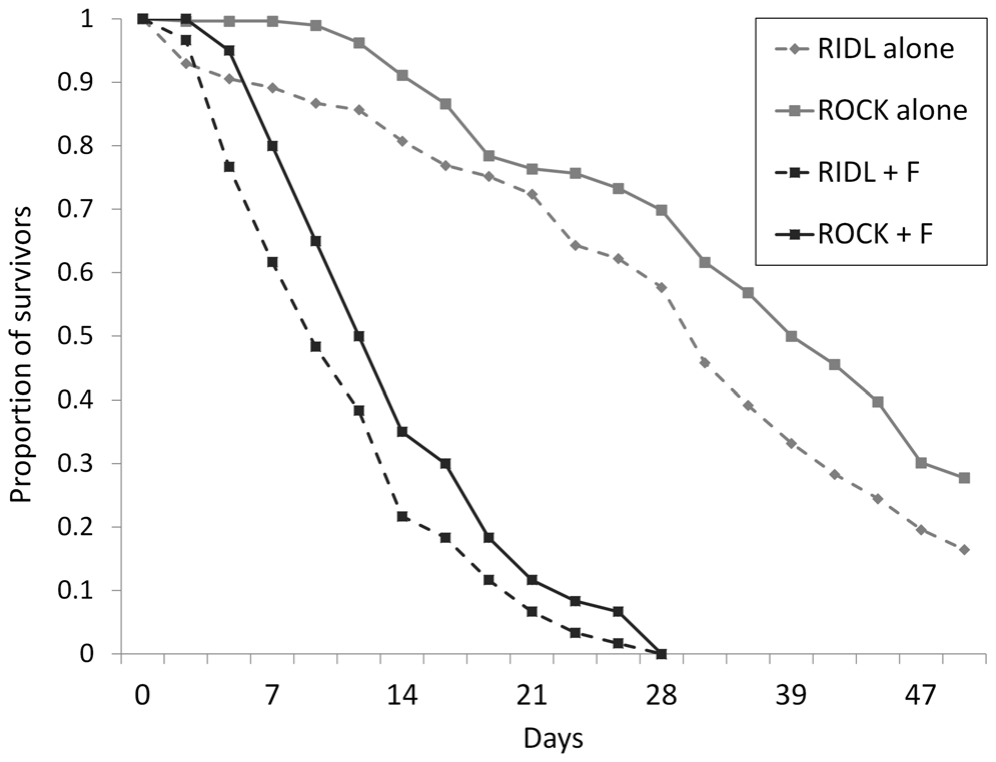
Lifespan of RIDL and ROCK males. Proportions of *Ae. aegypti* males surviving when (i) 150 males (RIDL or ROCK) were caged alone (grey lines) or (ii) when 30 males (RIDL or ROCK) were caged with 120 ROCK females (black lines). Data are the average of two replicates for each experiment (males with or without females). The LT_50_ values ( = median longevity values) were 39 for ROCK males alone, 32 for RIDL males alone, 11 for ROCK males caged with females and 9 for RIDL males caged with females. All mosquitoes were maintained off-TET.

Longevity (LT_50_) was higher when males were maintained without females. The median longevity of RIDL males was approximately 18% lower than that of ROCK males whether held with or without females (Figure 3). For males only, there was a significant difference in longevity between replicates for RIDL males (Log-rank test, p = 0.0014) but not for ROCK males (Log-rank test, p = 0.38). The RIDL vs. ROCK difference, when differences for replicates within types of males were allowed for, were significant for both the raw data (GLM, p<0.0001) and log-transformed data (GLM, p<0.0001). However, the assumption of normality for both GLMs was not valid (Shapiro-Wilk, p<0.0001). In this experiment, there is evidence that RIDL males have a reduced longevity if differences between cages are ignored (Log-Rank, p = 0.0004; Wilcoxon, p<0.0001; PH likelihood, p = 0.001).

For males with females, there were no significant differences between replicates for RIDL males (Log-Rank, p = 0.29) and ROCK males (Log-Rank, p = 0.42). However, the comparison RIDL vs. ROCK that ignored differences between replicates gave different results (Log-Rank, p = 0.060; Wilcoxon, p = 0.012; PH likelihood, p = 0.11). We therefore performed the same analysis as above. When differences for replicates within types of males were allowed for, the RIDL vs. ROCK differences were significant for both the raw data (p = 0.026) and log-transformed data (p = 0.006). However, the assumption of normality for both GLMs was not valid (Shapiro-Wilk: raw data, p = 0.001; log-transformed data, p = 0.0062). In this experiment, a significant difference between RIDL and ROCK male longevity is not clear.

### Anomalous survival of heterozygous RIDL progeny reared off-TET

Of 4265 larvae hatched for this experiment, 788 (18.4%) survived to adulthood. Of these, 104/350 (29.7%) females and 195/438 (44.5%) males survived for one week or more. Thus, surviving one-week old adults represented about 7% (2.5% females and 4.5% males) of hatched larvae compared to 99.5% of ROCK larvae in the control. This rate was unexpectedly high, markedly greater than in published rates for this strain (3–5%, Phuc *et al.*, 2007 and unpublished data). Comparison of procedures revealed that whereas larvae in our studies were reared on a commercial cat food (Purina ONE®, Nestlée Purina PetCare France, Rueil-Malmaison, France), larvae in previous published and unpublished studies had been fed a brand of fish food widely used in mosquito insectaries (TetraMin®, Tetra GmbH, Melle, Germany). We therefore ran a side-by-side comparison of the two procedures. Of the 9847 larvae hatched and fed on cat food, 1818 (18%) survived to adulthood. Of the 10413 larvae hatched at the same time but fed on fish food, 402 (3.9%) survived to adulthood—consistent with the previous observations of Phuc *et al.* (2007).

## Discussion

Our studies demonstrated that the key aspects of the fitness of *Ae. aegypti* RIDL males carrying a tetracycline repressible lethal gene was comparable to that of ROCK males, an encouraging step towards the application of this transgenic strain and genetic control strategy. This conclusion is supported by recent field data showing that OX513A males can compete for mates with wild males in the field, and that sustained release can suppress a target field population of *Aedes aegypti* [29], [30].

### Fertilisation and oviposition

Nearly 100% of ROCK females in the mating competition experiments were fertilised, and there was only one case of mixed progeny (0.3%). This was presumably the result of a double fertilisation, though a similar outcome could be obtained if the RIDL strain contained heterozygotes. We have no reason to suspect this alternative explanation here, which would in any case not affect the interpretation. Monogamy [31], [32], or at least first-male paternity [33], is considered typical for *Aedes aegypti*, though Gwadz and Craig found that inadequate transfer of semen from male *Ae. aegypti* can result in females remating [34]. Helinski et al [35] revisited the question of polyandry in large field cages finding 14% of females had engaged in multiple matings. In laboratory mating tests similar to those described here, double mating has previously been observed by the authors at 0–6% of total inseminations (data not shown). Clearly, under certain conditions, *Ae. aegypti* females can fertilise eggs using sperm from more than one male. Nearly three times as many eggs were laid per female in the 2 ♂/1 ♀ experiment (mean = 120.3) than in the 10 ♂/5 ♀ experiment (mean = 42.3). This may have been due to a reduction in the quantity of blood ingested due to crowding. Alternatively, the sperm of RIDL males may be higher in quality and/or quantity because ROCK females fertilized by RIDL males laid more eggs than those fertilized by ROCK males. These data indicate that RIDL males are as effective as ROCK males at inducing refractoriness to remating in wild type females.

### Insemination capacity

The maximum number of females fertilized by RIDL and ROCK males was similar to that in other studies [36]–[38], and showed no reduction in the ability of RIDL males to inseminate multiple females, relative to wild type.

### Mating competitiveness

Andreasen and Curtis [39] found that *Anopheles stephensi* and *An. gambiae* males irradiated as adults were as competitive as non-irradiated males, but were less competitive when irradiated as pupae. In the 10 ♂/5 ♀ experiment, there was no difference between expected and observed frequencies of transgenic crosses, in other words no indication that the RIDL males were less competitive than wild type. However, in the 2 ♂/1 ♀ experiment, there were fewer transgenic matings than expected. The reason for these apparently contradictory results is not clear. Laboratory experiments of this type inevitably differ considerably from natural conditions. In the wild, females can probably choose between more than two males, so the 10 ♂/5 ♀ experiment may have been more ‘natural’ in that regard. On the other hand, the 0.54 l mating arena constrains the mosquitoes to a higher density than in the wild; this effect may be more pronounced for the 10 ♂/5 ♀ experiment.

### Adult male longevity

Irvin et al [24] found that one homozygous *Ae. aegypti* strain out of the three had reduced adult longevity but Moreira et al [25] found no significant difference in the survival of two heterozygous strains of *An. stephensi*. Note that when homozygous RIDL males are maintained off-TET, there should be an additional cost because adults express the lethal gene. The protocol used – larvae reared on-TET and adults held off-TET, was to mimic the conditions to which RIDL males would be exposed if reared and released into the field in a control program. In our study, the median longevity of newly-emerged RIDL males was slightly reduced (18%) relative to ROCK males. This modest reduction is similar to that seen for related molecular constructs in the Mediterranean fruit fly *Ceratitis capitata* (0, 13 and 21% reduction for three different transgenic lines [40]).

### Fitness

Fitness can be defined as the relative success of an individual in passing its genes to the next generation. For mosquitoes, it can be estimated as (i) survival, measured as larval biomass productivity, development time, adult emergence, larvae/adult survival, and (ii) reproduction, including parameters such as fecundity, fertility, mating competitiveness. We did not find significant differences between RIDL and ROCK in fecundity or mating capacity. In addition there was no significant difference in mating competitiveness when several RIDL males competed with several ROCK males. However, longevity of RIDL males was lower than for ROCK males and RIDL males were less competitive in the experiment 2 ♂/1 ♀.

The lower survival of RIDL males could be due to inbreeding, and/or the expression of the lethal gene during adult stage, as adults were off-TET. In addition, the RIDL strain expresses the DsRed2 protein under the control of the ubiquitous Actin5C promoter. Ubiquitous promoters may have a stronger impact on fitness than tissue- or stage-specific promoters [22].

### Tetracycline contamination

Large quantities of tetracycline and other antibiotics are used to boost growth in factory-reared chickens. The label on the cat food that we used states “made with selected chicken” and the list of ingredients included a minimum of 16% chicken plus dehydrated poultry protein, hydrolysed liver (source not specified, possibly chicken) and “animal fats”, ingredients that are derived from processed poultry offal and bone-meal. By contrast, TetraMin® comprises fish, molluscs, crustaceae and vegetable materials presumably free of tetracycline contamination.

Studies have shown that poultry products (even those used for human consumption) may contain oxytetracycline, tetracycline and chlortetracycline [41]–[43] at relevant concentrations (e.g. 1–2 µg/ml [42]) and there is little doubt that the presence of such compounds gave rise to the anomalous survival of insects reared on cat food. In nature it is highly unlikely that larvae of *Ae. aegypti* would ever be contaminated with tetracycline because it is a container-breeding species, not present in ground pools or other sites where contamination with tetracycline is possible; in its original habitat, it breeds in tree-holes and other natural containers but it has adopted the urban, peri-domestic environment by breeding in artificial containers — discarded tyres, buckets and cans, flower pot saucers etc. — hence its importance as a highly effective urban vector of yellow fever, chikungunya and dengue.

In summary, the mating competitiveness of a strain of *Ae. aegypti* with a late-acting, tetracycline-repressible gene was comparable to that of the ROCK strain. Our results encouraged us to continue our studies in more realistic settings, and using a more wild-type genetic background. We introgressed the OX513A insertion into a Mexican-derived strain and found this derivative to have good mating competitiveness in the field [30] and indeed used it to suppress a target field population of *Ae. aegypti* [29]. In addition, we are investigating dispersal and survival of male and female mosquitoes in the field. Such data are essential to optimise control strategies and field releases in the future. As we have shown that late-acting RIDL insects have a comparable fitness as the ROCK strain from which it was produced, this adds to the growing evidence that transgenic mosquitoes can be produced without gross effects on fitness.

## Methods

### Mosquito strains

Throughout this report, transgenic OX513A mosquitoes homozygous for the RIDL construct are referred to as “RIDL” unless mentioned otherwise. In addition, rearing of strains with or without tetracycline (TET) are referred to as “rearing on/off-TET” and maintenance of adults without TET in the sugar water are referred to as “off-TET”.

All experiments were conducted with the RIDL and ROCK strains. Larvae, ca. 250–300 per tray (20×30 cm, 1.5 litres), were fed on commercial chicken-based cat food (Purina ONE®), except as noted. Larval density was maintained at ca. 250–300 larvae per tray. For rearing of the RIDL strain, 30 mg/litre of tetracycline hydrochloride (Sigma®) was added to the rearing water, and to the 10% sugar water and blood offered to adults. Insectaries were maintained at 26°C (±1°C) and 60% (±10%) relative humidity with 12-hour light/dark cycle. All data were analysed with the software package SPSS version 13 (SPSS Inc., Chicago, IL). In addition, the SAS System for Windows (8.02) was used for adult male longevity analyses.

### Mating competitiveness

ROCK larvae were reared off-TET, RIDL larvae were reared on-TET and pupae transferred to individual 15 ml plastic tubes. Males and females were separated by gender and caged for 2–3 days to attain sexual maturity. Mating experiments were conducted in cardboard cylindrical cages (diameter 8.5 cm, height 9.5 cm, volume 539 ml) for seven days. As adults, all mosquitoes were maintained off-TET.

Mosquitoes were introduced to the cylinder cages after immobilization at 4°C. Females were introduced after males. Females were offered off-TET heparinated (1000 IU/ml) rabbit blood via a Parafilm® membrane [44] after seven days. Males were removed right after blood feeding. One day after blood feeding, females were transferred into individual cages. Eggs were collected on wet cotton disks (make-up removers) and dried for at least 3 days in the laboratory before hatching. Spermathecae of females that did not oviposit were examined for sperm. Eggs were submerged for 48 hours and larvae reared off-TET no more than 1 month after egg laying. First or second instar larvae were transferred to individual wells of 96-well plates and screened for DsRed2 fluorescence. Mortality of the larvae was recorded.

#### Mating competitiveness: 2 ♂/1 ♀

We compared mating success of two males paired with one ROCK female. Three sets of 60 cages, each with one ROCK female, contained either (i) two ROCK males, (ii) two RIDL males or (iii) one RIDL male and one ROCK male. Survival of the adults was monitored daily. Cages containing dead mosquitoes were eliminated. Eggs were hatched off-TET and mortality was recorded. Including all the data, we compared the number of eggs in transgenic crosses (RIDL male) to non-transgenic crosses (ROCK male) using the Mann-Whitney test. After removing the RIDL and ROCK controls from the dataset, we tested whether the observed frequencies of transgenic and non-transgenic crosses differed from the expected (equal frequency, based on a null hypothesis of equal competitiveness of the two male genotypes) using the χ^2^ test.

#### Mating competitiveness: 10 ♂/5 ♀

We assessed the mating competitiveness of RIDL vs. ROCK males when caged with five ROCK females, using a range of males of the two strains (ratio of strains ROCK/RIDL: 10/0, 8/2, 5/5, 2/8, 0/10), with eight replicates of each. We recorded the number of eggs, the number of larvae hatching off-TET, the mortality of the resulting pupae. All surviving pupae were transferred to tubes and sexed if they reached adulthood. We compared the number of eggs in transgenic to non-transgenic crosses using the Mann-Whitney test. We analysed the difference between the numbers of eggs laid across the different ratio of strains using the Kruskal-Wallis test. Lastly, we compared the expected frequencies of transgenic and non transgenic crosses with the observed frequencies, for each type of crosses, using the χ^2^ test. The expected probabilities of a transgenic cross ranged from 0 (10 ROCK/0 RIDL), 0.2 (8 ROCK/2 RIDL), 0.5 (5 ROCK/5 RIDL), 0.8 (2 ROCK/8 RIDL) to 1 (0 ROCK/10 RIDL).

### Insemination rate

We tested whether RIDL and ROCK males could fertilise equal numbers of ROCK females. RIDL larvae were reared on-TET and RIDL and ROCK adults were maintained off-TET. Newly emerged adults were separated by gender and aged in cages for three to four days. Two experiments were performed, with 10 replicates of each: (i) one male (either RIDL or ROCK) caged with 15 females for 24 h and (ii) one male (either RIDL or ROCK) caged with 15 females until male's death. When experiments ended, females were killed by freezing and assessed for the presence of sperm. We compared the insemination of RIDL and ROCK males by analysing (i) the number of fertilized females and (ii) the number of spermathecae containing sperm using the Mann-Whitney test.

### Adult male longevity off-TET

We tested for variation in the longevity between RIDL and ROCK males when kept (i) with ROCK females or (ii) without. RIDL larvae were reared on-TET and RIDL and ROCK adults were maintained off-TET. We set up two replicates, using 30×30×30 cm cages, of each of the following: (i) 150 RIDL males, (ii) 150 ROCK males, (iii) 120 ROCK females with 30 RIDL males, (iv) 120 ROCK females with 30 ROCK males. Dead adults were collected and counted every three days until the last male died. LT_50_ values ( = median longevity in days) were estimated. The Log-rank test, the Wilcoxon test and the Proportional Hazards (PH) likelihood test were used to compare longevity of RIDL vs. ROCK males alone, and with females; (i) between replicates in both experiments and (ii) between RIDL vs. ROCK for both experiments (with and without ROCK females). When differences between replicates were found, we used a generalised linear model (GLM) on (i) the raw data and (ii) the log-transformed data. The Shapiro-Wilk test was used to test for normality of GLM residuals.

### Survival of heterozygous RIDL progeny reared off-TET

Heterozygous RIDL eggs were hatched and reared off-TET. Larval, pupal and adult mortality was recorded. Surviving pupae were transferred to cages (30×30×30 cm) for recording pupal and adult mortality. 200 ROCK pupae were placed into another identical cage for control. One week after pupae were put into the cages, the number of surviving of adults was recorded.
